# Meta-analysis of serum C-reactive protein and cartilage oligomeric matrix protein levels as biomarkers for clinical knee osteoarthritis

**DOI:** 10.1186/s12891-018-1932-y

**Published:** 2018-01-19

**Authors:** Junfeng Zhang

**Affiliations:** Department of Health Statistics / the Publishing house, Public Health of Shanxi Medical University / Chinese Journal of Rheumatology, Taiyuan, Shanxi 030001 China

**Keywords:** C-reactive protein, Knee osteoarthritis, Pain, Meta-analysis

## Abstract

**Background:**

The roles of C-reactive protein (CRP) and cartilage oligomeric matrix protein (COMP) in knee osteoarthritis (KOA) remain controversial, thus the present study is aimed to explore the relationships between CRP, COMP, and the incidence/progression of KOA.

**Methods:**

A systematic search was conducted on PubMed and Embase until September, 2016 for all the relevant studies. The pooled mean difference (MD) with its 95% confidence interval (95% CI) based on fixed effects model or random effects model was calculated to assess the potential role of CRP and COMP in the incidence or progression of KOA. Heterogeneity was evaluated by Cochran’s Q and I^2^ tests. When *P* < 0.05 or I^2^ > 50%, a random effects model was chosen, otherwise, a fixed effects model was used. Moreover, the role of CRP in different degrees of pain was also analyzed. Sensitivity analysis was performed to evaluate the strength of the meta-analysis.

**Results:**

Fourteen studies were enrolled in the meta-analysis. No difference was found between baseline CRP and CRP levels in the last follow-up period of KOA (MD = − 0.09, 95% CI: -0.30, 0.13). Pooled data showed higher CRP concentration in patients with incident KOA when compared with controls (MD = 0.33, 95% CI: 0.04, 0.63). Moreover, higher serum COMP levels were found in patients with incident KOA (MD = 1.69, 95% CI: 0.61, 2.76) Additionally, significant higher CRP concentration was observed in KOA patients with highest degree of pain (MD = 1.60, 95% CI: 0.52, 2.67).

**Conclusion:**

CRP and COMP serum levels were both associated with the incidence of KOA. Patients with a higher CRP and COMP concentration might have an increased probability of developing KOA. However, higher CRP serum levels was not related with KOA progression. Furthermore, KOA patients with more pain had higher CRP concentrations.

## Background

Osteoarthritis (OA), the most common type of arthritis, is characterized by joint space deterioration, pain, and loss of motion. Epidemiology studies have observed high prevalence of OA both in developed countries and developing countries. For example, the overall prevalence of knee OA was found to be 28.7% in India [1], the prevalence was highest (13.7%) in subjects living in the South-West region in China [2], and OA affected nearly 27 million Americans [3]. Additionally, the prevalence and incidence of the disease continue to increase. OA can become both a financial and health burden to patients and can affect their quality of life. Therefore, it is urgent to find an effective way to diagnosis and prevent the incidence or progression of OA in the early stage of disease.

Inflammation plays an important role in the pathogenesis of OA, although the specific correlations between biomarkers of inflammation and knee osteoarthritis (KOA) remain controversial. C-reactive protein(CRP) is a pentameric protein found in blood plasma associated with inflammation, infection, and injury. Studies demonstrated that CRP was correlated with complications, such as hypertension, cardiovascular disease, and diabetes [4, 5]. Studies have suggested that KOA was significantly related with CRP concentration [6, 7]. Other studies suggested that there were significant association between CRP concentration and KOA incidence [8–10]. Cartilage oligomeric matrix protein (COMP) is a 535-kDa non-collagen protein which has strong relation with the incidence of KOA. Some evidence has demonstrated that COMP serum levels in OA was different from healthy controls.

Because of these conflicting reports, the purpose of this study was to evaluate the association between CRP concentration, COMP serum levels and the incidence/progression of KOA. The relationship between CRP concentration and pain in KOA patients was also assessed.

## Methods

### Search strategy and study selection

A systematic search was performed on PubMed and Embase from database inception through September 2016. The following search terms were used: “osteoarthritis”, “knee osteoarthritis”, “C-reactive protein”, “CRP”, “cartilage oligomeric matrix protein”, “COMP”. Search terms were combined with OR and AND. All the reference list of retrieved studies and any additional publication were also searched and screened. The study was conducted based on the PRISMA guidelines [11].

### Inclusion criteria

The included studies were in line with the following criteria: 1) subjects had clinical KOA; 2) investigated the association between KOA incidence and progression and the concentration of CRP or COMP serum levels; 3) parameter of CRP or COMP could be extracted or calculated; 4) published in English.

We excluded studies in which KOA patients were formerly treated by medication. In addition, repeated studies, reviews, comments, and letters were also excluded.

### Data extraction and quality assessment

Data were extracted by two independent researchers following pre-designed form. The quality assessment was performed independently by two reviewers, and disagreement would be solved by the third reviewer.

Baseline characteristics of the eligible studies included first author, publication year, patients’ demographic, study design, research area, the concentration of CRP and COMP serum levels, and the number of the number of KOA and control patients. The quality of the studies was assessed by Newcastle-Ottawa Scale (NOS) [12]. In brief, NOS contains 8 items, categorizes into three groups, and with a total score of 9 stars. Each eligible study was graded according to this tool. In the current study, a cohort study with a follow-up rate > 75% was assigned one star. If NOS score of the study reached more than 5, it was considered as high quality literature, and was suitable for the meta-analysis.

### Statistical analysis

We extracted mean difference(MD) and standard deviation(SD) of CRP and COMP levels at baseline and at the end of the follow-up period of study subjects and controls. Heterogeneity among the included studies was calculated by Cochran’s Q-test and I^2^ test [13]. *P* < 0.05 or I^2^ > 50% was defined as significant heterogeneity between individual studies. Then, the random effects model was selected for pooling MD and SD of CRP and COMP levels to assess the association between CRP and COMP concentration and KOA incidence and progression. When *P* > 0.05 or I^2^ < 50%, a fixed effect model was chosen to pool MD and SD of CRP and COMP levels. Sensitivity analyses were performed by changing one study at a time, to see what effect this produces on the outcome. All statistical analysis was performed by Cochrane Collaboration Review Manager (version 5.3). We considered *P* < 0.05 as statistically significant.

## Results

### Search results and study selection

**Fig. 1:**
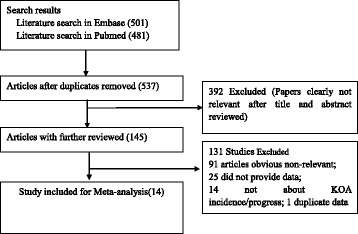
Flowchart of study selection for inclusion in the systematic review

As shown in Fig. 1, the initial search yielded a total of 982 articles. 537 remained after removing the duplicated articles. 392 studies were further excluded because they did not investigate the relationship between CRP or COMP concentration and the incidence/progression of KOA. A total of 145 articles were under full-text review, of which 131 studies were excluded. Among the 131 articles, 91 articles were reviews, comments or case reports, 25 studies did not provide sufficient data, 14 studies were not about KOA incidence or progression, and 1 duplicate population [9, 14]. Eventually, 14 articles were included in the meta-analysis [6, 7, 14–25].

All relevant baseline characteristics of the enrolled studies were summarized in Table 1. In general, nine studies were designed as case control, and five were designed as cohort studies. Four studies were conducted in Asia, seven were performed in Europe, one was in Australia, and two were in USA. Study sizes ranged from 15 to 5171 patients, and the follow-up period ranged from 2 weeks to 12.4 years. Furthermore, all the included studies were scored more than 5 stars based on the NOS criteria, suggesting all enrolled studies were suitable for meta-analysis.

**Table 1:** Baseline chacteristics of enrolled studies

Study	Design	Number	Age (years)	Follow-up	area
Fernandes, 2007	Case control	KOA:75 Control: 40	KOA:56.6 ± 7.6; Control: 53.8 ± 8.5	NA	Brazil
Senolt, 2005	Case control	KOA:38 Control: 38	KOA:64.1 ± 10.1; Control: 58.3 ± 9.1	NA	USA
Wakitani, 2007	Case control	KOA:31 Control: 24	KOA:20–80; Control: 23–52	NA	Japan
Li, 2012	Case control	KOA:115 Control: 35	KOA:55 ± 13.2; Control:53 ± 12.53	2–3 years	China
Engstrom, 2009	population-based cohort	5171	57.5 + 5.9	12.4 years	Sweden
Zhang, 2015	Case control	Control: 20 KOA: 20	Control:56.3 ± 7.9; KOA: 59.2 ± 8.3	NA	China
Sowers, 2002	cohort	1025	42.9 ± 0.1	2.5 years	USA
Kerkhof,2010	Case control	KOA: 210; Control: 1360	≥55 years	NA	Netherlands
Haghighian,2014	Case control	23	58.27 ± 7.84	2 months	Iran
Ramirez, 2014	cohort	186	61 ± 7.3	2 years	Netherlands
Nielsen, 2014	Case control	281	40–80	NA	Denmark
Scorei, 2011	Case control	15	67.6 (5.5)	2 weeks	Romania
Stannus, 2012	cohort study	KOA:35; Control:114	KOA:63.2 ± 6.9 Control: 61.5 ± 6.7	5 years	Australia
Hosnijeh, 2016	population-based prospective cohort	1335	65.2 (8.43)	5-year	Netherlands

*KOA* knee osteoarthritis

### Meta-analysis of the association between KOA progression and CRP levels

**Fig. 2:**
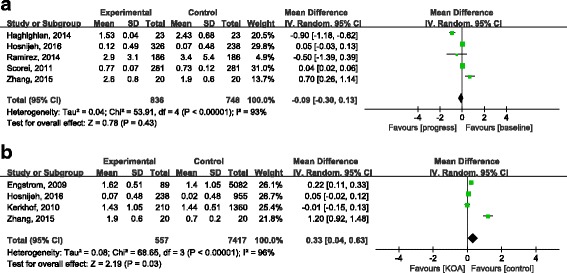
Pooled effect size of the relationship between C-reactive protein (CRP) concentration and knee osteoarthritis (KOA). **a** the association between CRP concentration and KOA progression; **b** the association between CRP concentration and KOA incidence

As shown in Fig. 2a, 5 studies including 836 KOA patients at baseline and 748 patients at the end of the follow-up period evaluated the association between KOA progression and CRP levels [6, 16–18, 21]. Significant heterogeneity was observed among the five studies (*P* < 0.00001, I^2^ = 93%). Therefore, a random effects model was used to pool data. No difference was found between baseline CRP and CRP levels at the last follow-up period in KOA patients (MD = 0.09, 95%CI: -0.30, 0.13). Because there was significant heterogeneity among the studies, sensitivity analysis was performed, and no discrepancy results were observed when we omitted one study at a time (Table 2).

**Table 2:** Sensitivity analysis for the meta-analysis

	Outcomes	No. of studies	MD(95% CI)	Heterogeneity test	
				*P*	I^2^ (%)
KOA progress with CRP	All studies	5	0.09 [−0.30, 0.13]	< 0.00001	93.0
	Haghighian, 2014	4	0.07 [− 0.04, 0.18]	0.02	70.0
	Hosnijeh, 2016	4	−0.15 [− 0.77, 0.47]	< 0.00001	94.0
	Ramirez, 2014	4	−0.07 [− 0.29, 0.16]	< 0.00001	94.0
	Scorei, 2011	4	−0.15 [− 0.78, 0.49]	< 0.00001	94.0
	Zhang, 2015	4	−0.20 [− 0.42, 0.02]	< 0.00001	93.0
KOA incidence with CRP	All studies	4	0.33 [0.04, 0.63]	< 0.00001	96.0
	Engstrom, 2009	3	0.39 [−0.08, 0.86]	< 0.00001	97.0
	Hosnijeh, 2016	3	0.45 [−0.05, 0.95]	< 0.00001	97.0
	Kerkhof, 2010	3	0.46 [0.06, 0.86]	< 0.00001	97.0
	Zhang, 2015	3	0.09 [−0.03, 0.21]	0.01	76.0
KOA pain with CRP	All studies	3	1.60 [0.52, 2.67]	0.001	85.0
	Nielsen, 2014	2	1.69 [−0.02, 3.40]	0.03	78.0
	Sowers, 2002	2	1.13 [0.44, 1.82]	0.49	0.0
	Stannus, 2012	2	1.88 [0.76, 3.00]	0.003	89.0

*KOA* knee osteoarthritis, *CRP* C-reactive protein

### Meta-analysis of the association between KOA incidence and CRP levels

Four studies including 557 KOA patients and 7417 controls were enrolled in the meta-analysis to assess the relation between KOA incidence and CRP levels (Fig. 2b) [7, 14, 17, 21]. A random effects model was selected due to significant heterogeneity among studies (*P* < 0.00001, I^2^ = 96%). The pooled MD with its 95% CI suggested that CRP concentration was somewhat higher in KOA patients when compared with healthy controls (MD = 0.33, 95% CI: 0.04, 0.63). High heterogeneity also existed in this subgroup. Therefore, sensitivity analysis was conducted by deducting one study at a time. When we removed the study by Engstrom et al., Hosnijeh et al., and Zhang et al., pooled data suggested no significant difference in CRP concentration between KOA patients and healthy controls (Table 2).

### Meta-analysis of the association between COMP serum levels and KOA incidence

**Fig. 3:**
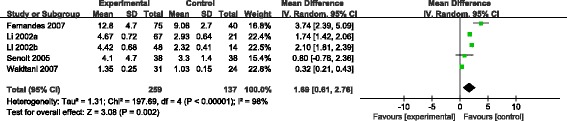
Pooled effect size of the relationship between cartilage oligomeric matrix protein (COMP) serum levels and knee osteoarthritis (KOA)

Figure 3 shows the association between KOA incidence and COMP serum levels from 5 studies containing 259 KOA patients and 137 healthy controls. Pooled data indicated that patients with clinical KOA had a higher serum COMP levels than controls (MD = 1.69, 95% CI: 0.61, 2.76). Sensitivity analysis was performed to evaluate the strength of this conclusion, and no inconsistent results were found when we omitted each study (Table 2).

### Meta-analysis of the association between CRP levels and pain in KOA patients

**Fig. 4:**
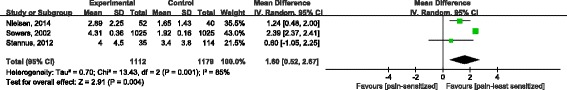
Pooled effect size of the relationship between C-reactive protein (CRP) concentration and pain in knee osteoarthritis (KOA)

As shown in Fig. 4, three studies estimated the correlation between CRP levels and pain in KOA patients [15, 19, 20]. A total of 2291 patients were included, among them 1112 patients reported knee pain at baseline assessment, and 1179 patients without knee pain were set as controls. Higher mean concentrations of CRP were found in patients who self-reported the highest level of knee pain (MD = 1.60, 95% CI: 0.52, 2.67). When we removed the study by Nielsen et al. in the sensitivity analysis, the significant difference between groups disappeared.

## Discussion

For inflammatory diseases, C-reactive protein (CRP) concentration is recognized as the most common laboratory marker, several clinical studies put forward that CRP concentration was elevated in patients with KOA [26]. Additionally, COMP serum levels are also considered as a potential biomarker for the diagnosis of KOA. The present meta-analysis demonstrated that higher CRP concentration was associated with higher KOA incidence, however, CRP levels were not related to the progression of KOA. Moreover, CRP concentration was higher in KOA patients with higher degrees of knee pain. Higher serum COMP levels in patients with incident KOA were also observed. Thus, both CRP and COMP could serve as useful biomarkers of KOA.

The outcome of our study suggests that CRP as a biomarker plays a positive role in the diagnosis and prognosis of KOA. This finding is consistent with a previous study [27]. However, in sensitivity analysis, when removing the study by Engstrom et al. [6], Hosnijeh et al. [17], and Zhang et al. [21], the significant difference of CRP levels between KOA patients and control group diminished. Thus, further study enrolling larger sample size and balancing confounding factors such as patient’s backgrounds is needed. Although significantly higher COMP serum levels was observed in incident KOA, no stratified analysis based on severity of KOA was performed due to limited enrolled studies. Further investigation is required to determine if this marker can be utilized to assess the incidence OA. Given that both CRP and COMP levels were higher in patients with KOA, the two factors could be used in combination for the prognosis of KOA.

Significant heterogeneity existed in our study. Although we conducted sensitivity analysis to assess the strength of the meta-analysis using one-at-a-time method, heterogeneity did not reduce. In patients with early KOA, many factors could affect the concentration of CRP, such as life habit and the background of KOA. For example, a previous study indicated that cigarette smoking was positively associated with serum CRP levels [28]. In addition, experimental data suggested both dietary and serum Mg were inversely related with serum CRP in early radiographic KOA patients [29]. Moreover, serum CRP was associated with knee bone marrow lesions scores [30]. Thus, future study stratified by living habit and background of patients should be performed to verify the present conclusion.

There are some limitations should be noted in the meta-analysis. First, the number of enrolled subjects was small, which limited the subgroup analysis stratified by smoking status, and other confounding factors such as hyperlipidemia. Second, we did not restrict the timing of biomarker assessment, and exercise status of the patients, also the follow-up period ranged from 2 weeks to 12.4 years, which might be one of the reasons causing significant heterogeneity among individual studies. Third, a number of studies did not provide calculated data. Moreover, other chronic diseases might influence the CRP levels in patients, and we did not eliminate the effect of those factors. Our study was designed to evaluate the relationship between biamarkers and KOA. The association between CRP and other OA types, such as hand, hip, and spine OA was not considered.

## Conclusion

The present meta-analysis indicated that both CRP and COMP could serve as biomarkers for KOA. Higher CRP and COMP serum levels might be associated with a higher incidence of KOA. However, higher CRP concentration was not associated with the progression of KOA. Further study is required verify the actual relationship between CRP and KOA incidence.

## Abbreviations

CI: Confidence interval
COMP: Cartilage oligomeric matrix protein
CRP: C-reactive protein
KOA: Knee osteoarthritis
MD: Mean difference
OA: Osteoarthritis
